# Can artificial intelligence with multimodal imaging outperform traditional methods in predicting age-related macular degeneration progression? A systematic review and exploratory meta-analysis

**DOI:** 10.1186/s12911-025-03119-z

**Published:** 2025-09-01

**Authors:** Kai-Yang Chen, Hoi-Chun Chan, Chi-Ming Chan

**Affiliations:** 1 Department of General Medicine, Chang Gung Memorial Hospital (Linkou branch), Taoyuan, Taiwan; 2 School of Pharmacy, China Medical University, Taichung, Taiwan; 3 Department of Ophthalmology, Cardinal Tien Hospital, New Taipei City, Taiwan; 4 School of Medicine, Fu Jen Catholic University, New Taipei City, Taiwan

**Keywords:** Age-related macular degeneration, Artificial intelligence, Deep learning, Predictive models

## Abstract

**Purpose:**

Age-related macular degeneration (AMD) is a leading cause of irreversible vision loss, and its prevalence is expected to rise with aging populations. Early prediction of AMD progression is critical for effective management. This systematic review and meta-analysis evaluate the accuracy, sensitivity, and specificity of artificial intelligence (AI) algorithms in in detecting and predicting progression of AMD.

**Methods:**

Following the Preferred Reporting Items for Systematic Reviews and Meta-Analyses (PRISMA) guidelines, a systematic review and meta-analysis were conducted from inception to February 7th, 2025. We included five studies that assessed the performance of AI algorithms in predicting AMD progression using multimodal imaging. Data on accuracy, sensitivity, and specificity were extracted, and meta-analysis was performed using Comprehensive Meta-Analysis software version 3.7. Heterogeneity was assessed using the I² statistic.

**Results:**

Of the five studies, AI models demonstrated superior accuracy (mean difference: 0.07, 95% CI: 0.07, 0.07; *p* < 0.00001) and sensitivity (mean difference: 0.08, 95% CI: 0.08, 0.08; *p* < 0.00001) compared to retinal specialists. Specificity also showed a minimal but significant advantage for AI (mean difference: 0.01, 95% CI: 0.01, 0.01; *p* < 0.00001). Importantly, heterogeneity was minimal to absent across all analyses (I² = 0–0.42%), supporting the reliability and consistency of pooled findings.

**Conclusion:**

AI algorithms outperform retinal specialists in predicting AMD progression, particularly in accuracy and sensitivity. These findings support the potential of AI in AMD prediction; however, given the limited number of included studies, the results should be interpreted as exploratory and in need of validation through future large-scale, prospective studies.

**Supplementary Information:**

The online version contains supplementary material available at 10.1186/s12911-025-03119-z.

## Key messages

**What is known**

- AI algorithms have demonstrated potential in predicting AMD progression using multimodal imaging.
- Early prediction of AMD progression is crucial for effective management and treatment.

**What is new**

- AI models outperform retinal specialists in accuracy and sensitivity in predicting AMD progression.
- AI also shows a minimal advantage in specificity over retinal specialists.

**Supplementary Information:**

The online version contains supplementary material available at 10.1186/s12911-025-03119-z.

## Introduction

Age-related macular degeneration (AMD) is the leading cause of permanent vision loss worldwide, predominantly affecting elderly individuals [1]. AMD is classified into three distinct stages: early, intermediate, and late, with the late stage further divided into geographic atrophy (GA) and neovascular AMD (nAMD) [2]. The prevalence of AMD is expected to rise significantly due to an aging population and increased life expectancy, making it a growing public health concern. To prevent severe vision loss and achieve optimal treatment outcomes, early detection and accurate prediction of AMD progression are crucial [3].

Multimodal imaging plays a central role in AMD management by providing detailed visualization of retinal structures and biomarkers. The primary imaging modalities used in AMD detection include optical coherence tomography (OCT), fundus photography, optical coherence tomography angiography (OCTA), fluorescein angiography (FA), and autofluorescence imaging (AF) [4]. These imaging techniques generate complementary data, enhancing disease evaluation and improving predictions of disease progression. However, the interpretation of these images traditionally relies on clinical expertise, which can be subjective and time-consuming. One of the earliest efforts that demonstrated the potential of multimodal imaging for AI-based AMD diagnosis was conducted by Burulina et al. [5], who explored the integration of OCT and fundus images in a preliminary deep learning experiment. Although conducted on a smaller dataset, their study suggested that multimodal inputs could enhance diagnostic accuracy, laying the groundwork for subsequent advances in this domain.

Artificial intelligence (AI), particularly through machine learning (ML) and deep learning (DL) techniques, has revolutionized ophthalmology by automating image analysis and enabling predictive analytics. AI models can perform segmentation, feature extraction, and disease progression prediction with high accuracy and reliability [6]. In particular, DL algorithms have demonstrated superior performance compared to traditional clinical benchmarks in predicting AMD progression using OCT and fundus images. Despite these advancements, research on AI-based AMD evaluation through imaging has been inconsistent, with variations in methodology and performance assessment [7].

Recent studies have highlighted the potential of AI to enhance early detection and personalized treatment strategies for AMD. AI’s ability to analyze vast amounts of imaging data, identify subtle patterns, and predict disease progression before clinical signs become apparent presents an exciting opportunity for improving patient outcomes [8]. This shift toward more precise and proactive care is particularly relevant as AMD continues to impose a significant burden on healthcare systems globally. However, the integration of AI into clinical practice still faces challenges, such as the need for large, diverse datasets to train AI models, the need for clinician trust in AI outputs, and concerns regarding the generalizability of AI models across different patient populations and imaging technologies [9].

Furthermore, while multimodal imaging allows for a more comprehensive understanding of AMD, the optimal combination of imaging modalities for accurate disease prediction remains unclear. Different imaging techniques capture distinct aspects of retinal health, from structural changes to vascular abnormalities, which may influence AMD progression [10]. As a result, it is essential to examine how AI models leverage these multimodal datasets to improve prediction accuracy and whether certain combinations of imaging modalities outperform others. The evolution of AI algorithms, especially those capable of integrating diverse imaging inputs, has the potential to overcome limitations associated with single-modality assessments, offering a more holistic view of disease status and progression [11].

Despite the promising role of AI in AMD management, challenges remain in validating AI-based models for clinical use. Several factors, such as model transparency, the interpretability of predictions, and real-world application across varied patient demographics, must be addressed to ensure AI tools provide actionable insights in diverse clinical settings. In addition, collaboration between AI developers, clinicians, and regulatory bodies will be crucial to establish guidelines for the safe and effective use of AI in AMD diagnosis and treatment [12].

This systematic review and meta-analysis aims to synthesize evidence from peer-reviewed studies to assess the performance of AI algorithms using multimodal imaging in predicting AMD progression. By comparing AI-based methods with traditional or non-AI-based predictive approaches, this study seeks to determine their accuracy, sensitivity, and specificity in detecting advanced AMD features and/or predicting disease progression, ultimately informing clinical practice and future research. Additionally, this review provides a comprehensive evaluation of the current landscape of AI in AMD progression prediction, examining its strengths, limitations, and future directions for research and clinical adoption.

## Methods

### Study design

This systematic review was conducted following rigorous DTA review methods and reported in accordance with the PRISMA-DTA 2018 guidelines (Preferred Reporting Items for Systematic Reviews and Meta-Analyses of Diagnostic Test Accuracy Studies) [13]. The study aims to synthesize evidence from peer-reviewed literature to assess predictive accuracy, sensitivity, specificity, and overall clinical applicability, ultimately contributing to the standardization of AI methodologies in AMD management. Our systematic review has been registered on an online registration website, PROSPERO, the number is CRD42025644341.

### Search strategy

A comprehensive literature search was conducted across multiple electronic databases, including PubMed, Embase, Web of Science, Scopus, Cochrane Library, and Google Scholar. A comprehensive search was performed using all available data from inception to February 7th, 2025. The search strategy incorporated a combination of Medical Subject Headings (MeSH) terms and free-text keywords structured according to the PICOS framework. The population-related terms included “Age-Related Macular Degeneration,” “Macular Degeneration,” “Retinal Degeneration,” “Geographic Atrophy,” and “Neovascular AMD.” Terms related to intervention and technology focused on AI-based methodologies, including “Artificial Intelligence,” “Machine Learning,” “Deep Learning,” “Neural Networks,” and “Computer-Assisted Diagnosis.” The search also covered various imaging modalities such as “Multimodal Imaging,” “Optical Coherence Tomography (OCT),” “Fundus Photography,” “Fluorescein Angiography,” and “Autofluorescence.” The search was further refined by including outcome-related terms such as “Disease Progression,” “Prediction,” “Risk Assessment,” and “Prognosis.” To ensure relevance, studies published in English and peer-reviewed journals were included, and reference lists of identified articles were screened to capture additional relevant studies.

### Eligibility criteria

Eligibility criteria were defined based on the PICOS framework. The application of the PICOS framework enabled a methodologically robust and transparent selection process. By clearly delineating criteria across population, intervention, comparator, outcomes, and study design, we minimized heterogeneity related to study types, ensured relevance to clinical practice, and enhanced the replicability of our review. However, the specificity of these criteria—particularly the requirement for multimodal imaging and head-to-head performance comparisons—may restrict generalizability. For example, potentially informative studies using single-modality AI models or lacking comparator arms were excluded. As a result, the findings are most applicable to AI systems designed for multimodal workflows and may not generalize to all AI-driven diagnostic tools currently in development or use. Studies were included if they evaluated AI performance in either predicting progression to late-stage AMD (e.g., GA or nAMD) or detecting established features of late AMD that serve as clinical surrogates for progression. We recognize that some studies focused on detection rather than longitudinal prediction, but their inclusion was justified based on their relevance to severity classification and disease staging.

### Population

The population of interest included human participants diagnosed with AMD at any stage, regardless of age, gender, or ethnicity. Studies assessing the prediction of AMD progression, including progression to geographic atrophy or neovascular AMD, were considered. Exclusion criteria included studies on animal models, in vitro experiments, or those focusing on retinal diseases other than AMD.

### Intervention

Studies utilizing AI-based algorithms, such as machine learning or deep learning models, trained or validated using multimodal imaging data, were included. Studies using only traditional statistical models without AI components or those relying solely on single-modal imaging without integration of multiple modalities were excluded.

### Comparator

Comparator methods were essential for inclusion, requiring studies to compare AI models with other AI-based approaches, traditional clinical grading systems, or expert clinician assessments. Studies had to report performance metrics such as accuracy, sensitivity, specificity, and area under the curve (AUC) for comparative evaluation. Studies without a clear comparator or lacking quantitative performance metrics were excluded.

### Outcomes

Outcome measures of interest included predictive performance metrics such as accuracy, sensitivity, specificity, AUC, F1-score, and mean absolute error (MAE), as well as the clinical utility of AI models in predicting AMD progression. Studies that did not provide quantitative data on prediction performance or those with incomplete or unclear outcome data were excluded.

### Study design

Eligible study designs included peer-reviewed original research articles such as prospective or retrospective cohort studies, cross-sectional studies, and clinical trials. To ensure methodological rigor, only studies published in English with sufficient methodological detail for quality assessment and data extraction were included. Excluded studies comprised reviews, editorials, case reports, conference abstracts, and non-peer-reviewed articles. Studies with insufficient data for meta-analysis, such as those lacking performance metrics or sample sizes, along with duplicate publications or overlapping datasets, were also excluded. Studies that used overlapping datasets were carefully assessed. In principle, if multiple studies using the same dataset evaluated different AI models or diagnostic tasks, they would be eligible for inclusion in the qualitative synthesis.

### Data extraction and quality assessment

Data extraction and quality assessment were carried out systematically. Two independent reviewers (K.Y.C. and H.C.C.) screened titles, abstracts, and full texts to determine study eligibility. Any disagreements were resolved through discussion or, if necessary, adjudication by a third reviewer (C.M.C.). A standardized data extraction form was utilized to gather details on study characteristics, AI models, comparator methods, imaging modalities, and key outcome measures. During the data extraction and quality assessment phases, several methodological challenges were encountered. Many included studies lacked standardized reporting of diagnostic accuracy metrics, such as 2 × 2 contingency tables or pre-specified decision thresholds. To address this, we extracted all available performance indicators (e.g., AUC, sensitivity, specificity, accuracy) and used pooled mean difference calculations for comparison. Where necessary, corresponding authors were contacted for clarification. Variability in AI model architectures, prediction types (classification versus regression), and input modalities (e.g., monomodal vs. multimodal) further complicated data synthesis. These differences were managed through subgroup stratification and detailed documentation in summary tables.

To assess the risk of bias (ROB), two independent reviewers (K.Y.C. and H.C.C.) conducted evaluations, with discrepancies resolved through discussion with a senior research team member. Given that our review focused on studies evaluating diagnostic test accuracy, we applied the QUADAS-2 tool [14], which is specifically designed to assess risk of bias in diagnostic accuracy studies, covering domains such as patient selection, index test, reference standard, and flow and timing. Additionally, to account for comparative diagnostic evaluations between AI models and human assessors, we used QUADAS-C, an extension of QUADAS-2 tailored for comparative diagnostic accuracy studies. Although our protocol initially listed ROBINS-E, RoB 2.0, and ROBINS-I, we did not proceed with these tools as they are not optimal for DTA studies. This deviation from the initial plan was made to ensure methodological appropriateness and has been clearly described in the revised manuscript.

### Data synthesis and statistical analysis

our meta-analyses were constrained by the incomplete reporting of necessary diagnostic accuracy data. The case/control ratio was calculated as the number of subjects or images with the target condition divided by those without, using reported or estimated values from each study. We recorded the validation strategies employed by each study to assess model performance, including whether internal cross-validation, holdout test sets, or external validation cohorts were used. We now acknowledge that advanced meta-analytic techniques (e.g., bivariate or hierarchical summary receiver operator characteristic models) could not be applied due to these data limitations. The statistical analysis was conducted via Comprehensive Meta-Analysis software version 3.7; the meta-analysis was carried out with the help of a detailed method for the synthesis of data in the form of serious studies. Fixed-effects models were applied for outcomes with significant heterogeneity (I² >75%), accounting for variability across populations and methodologies, while fixed-effect models were used when heterogeneity was low. The mean difference (MD) with 95% Confidence Interval (CI) method was used to pool individual study results, and heterogeneity was assessed using the I² statistic, with p-values < 0.05 indicating significant heterogeneity. A Z-test determined the statistical significance of pooled outcomes, with *p* < 0.05 considered significant.

## Results

### Study selection

**Fig. 1:**
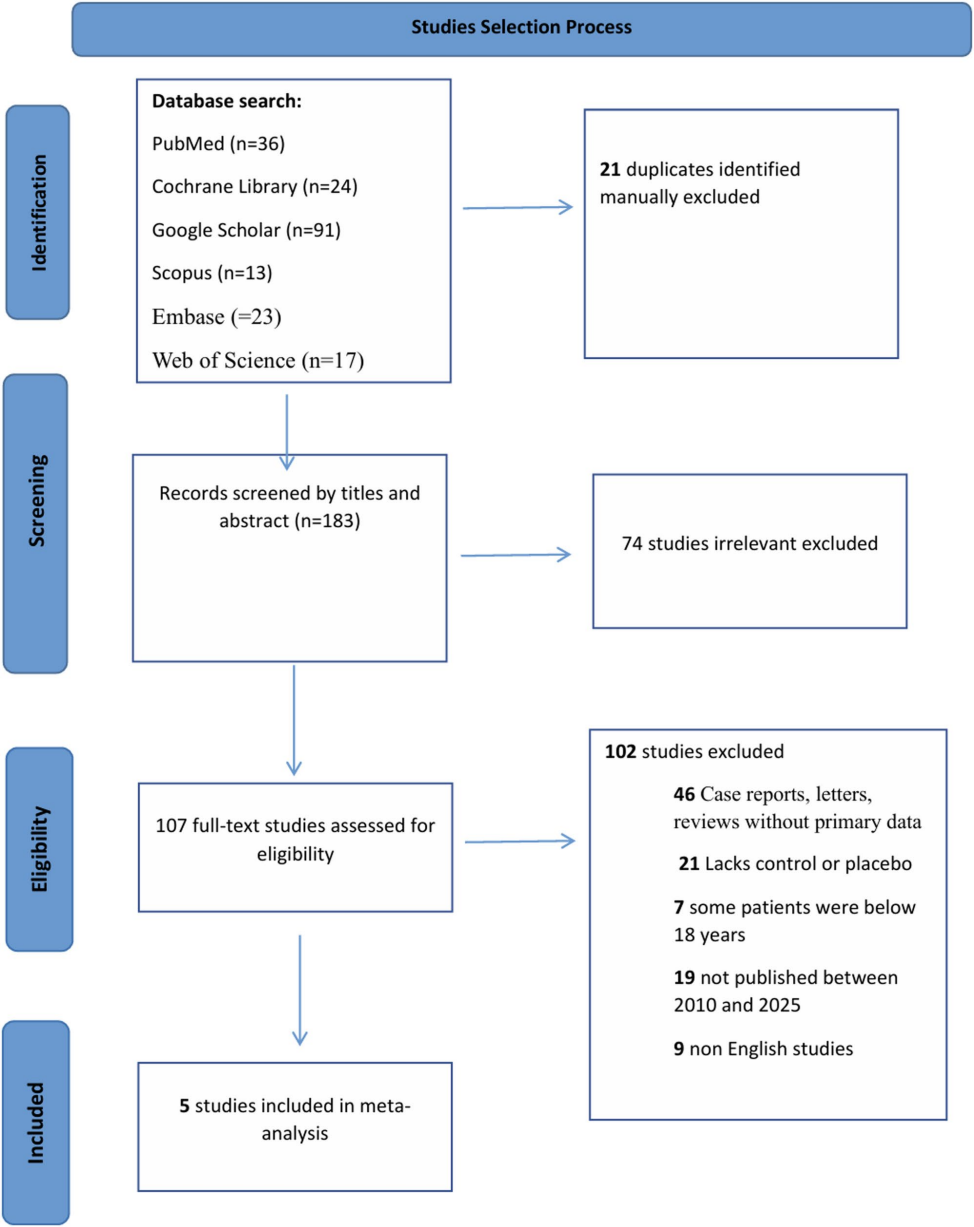
PRISMA flow diagram illustrating the study selection process for systematic review and meta-analysis

Figure 1 illustrates the study selection process for a meta-analysis, comprising four stages: identification, screening, eligibility, and inclusion. In the identification stage, studies were retrieved from six databases: PubMed provided 36 studies, the Cochrane Library yielded 24, Google Scholar contributed 91, Scopus added 13, Embase provided 23, and Web of Science supplied 17 studies. This search resulted in a total of 204 studies, but 21 duplicates were manually excluded, leaving 183 records for further screening. Although a total of 183 unique records were retrieved from six major databases, this reflects the novelty of the topic rather than an inadequacy in the search strategy. The field of AI-driven diagnostic assessment for AMD is still in its early stages, with relatively few peer-reviewed studies currently available. During the screening stage, the titles and abstracts of these 183 studies were reviewed, and 74 studies were excluded due to irrelevance and 2 records were not retrieved, reducing the number of studies to 107. At the eligibility stage, the full texts of these 107 studies were assessed. However, 102 studies were excluded for various reasons: 46 were case reports, letters, or reviews without primary data; 21 lacked a control group or placebo; seven included patients under 18 years of age; 19 were not published within the specified timeframe of 2010 to 2025; and nine were not published in English. Finally, in the inclusion stage, five studies met al.l eligibility criteria and were included in the meta-analysis [15–19]. No studies were found that used overlapping datasets to explore distinct AI approaches or diagnostic outcomes. Therefore, exclusion of overlapping studies was based on redundancy rather than methodological diversity.

### Baseline characteristics

**Table 1:** Characteristics of included studies

Study ID (Author and Year)	Study Design	Country	Sample	Participants age	AMD Stage	Total images	AI Algorithm model	Intervention	Comparator	Outcome Metrics/measures (Area under the curve (AUC), accuracy, sensitivity, specificity)	Key Findings	Case / Control Ratio
			Size									
Keenan et al., 2019	multi-center, prospective cohort study	United states	4,582 participants	55 to 80 years	No AMD to advanced AMD	59,812 color fundus photographs	Deep learning models (GA detection, CGA detection, and centrality detection)	AI-based detection of GA and CGA using fundus images	Human expert graders (retinal specialists)	GA Detection Model /Retinal specialists): Accuracy = 0.965 (95% CI: 0.959, 0.971) / 0.975 (95% CI: 0.971, 0.980), Sensitivity = 0.692 (95% CI: 0.560, 0.825)/ 0.588 (95% CI: 0.468, 0.707), Specificity = 0.978 (95% CI: 0.970, 0.985)/ 0.982 (95% CI: 0.978, 0.985)	AI-based GA detection showed high accuracy and was noninferior to human retinal specialists. AI models were also effective in detecting CGA.	0.045 (GA detection) and 0.025 (CGA detection)
										CGA Detection Model/Retinal specialists: Accuracy = 0.966 (95% CI: 0.957, 0.975)/ 0.990 (95% CI: 0.987, 0.993), Sensitivity = 0.763 (95% CI: 0.641, 0.885)/ 0.448 (95% CI: 0.255, 0.641), Specificity = 0.971 (95% CI: 0.960, 0.982)/ 0.993 (95% CI: 0.989, 0.996)		
										Centrality Detection Model/Retinal specialists: Accuracy = 0.762 (95% CI: 0.725, 0.799)/ 0.735 (95% CI: 0.445, 1.000), Sensitivity = 0.782 (95% CI: 0.618, 0.945)/ 0.878 (95% CI: 0.722, 1.000), Specificity = 0.729 (95% CI: 0.543, 0.916)/ 0.703 (95% CI: 0.332, 1.000)		
Zapata et al., 2020	Retrospective study	Spain	3,06,302	Not stated	Not stated	306,302 images	Three CNN architectures (two custom-designed)	Deep learning algorithms to classify retinal images and detect diseases	Model 1 (it control since it establishes a foundational capability to filter out irrelevant image types (CFP vs. OCT/Other))	-Model 1(Image type): AUC = 0.979 (95% CI: 0.978–0.981), Accuracy = 0.960 (95% CI: 0.957–0.962), Sensitivity = 0.977 (95% CI: 0.976–0.979), Specificity = 0.924 (95% CI: 0.920–0.929)	AI models effectively classify retinal images, evaluate quality, distinguish OD/OS, and detect AMD and GON with high performance	~ 1.0
										-Model 2 (Eye):		
										AUC = 0.989 (95% CI: 0.988–0.991), Accuracy = 0.974 (95% CI: 0.973–0.977), Sensitivity = 0.983 (95% CI: 0.981–0.985), Specificity = 0.966 (95% CI: 0.963–0.970)		
										-Model 3 (Image quality): AUC = 0.947 (95% CI: 0.945–0.948) 0.979, Accuracy = 0.918 (95% CI: 0.916–0.919), Sensitivity = 0.969 (95% CI: 0.968–0.970), Specificity = 0.818 (95% CI: 0.814–0.821)		
										-Model 4 (AMD):		
										AUC 0.936 (95% CI: 0.922–0.946), Accuracy 0.863 (95% CI: 0.845–0.877), Sensitivity 0.902 (95% CI: 0.880–0.916), Specificity 0.825 (95% CI: 0.799–0.849)		
										-Model 5 (GON): AUC = 0.863 (95% CI: 0.827–0.894), Accuracy = 0.803 (95% CI: 0.760–0.836), Sensitivity = 0.768 (95% CI: 0.710–0.824), Specificity = 0.838 (95% CI: 0.784–0.888)		
Ting et al., 2020	Validation cohort study	Singapore	14,880 patients	Mean age 60.2 years; 54.6% men	Early stages, Vision-threatening, Possible glaucoma, AMD	494,661 images for training; 71,896 images for validation	Deep Learning System (DLS)	Automated grading of DR, Glaucoma, AMD	Professional graders (retinal specialists, ophthalmologists, optometrists)	Experimental arm: Sensitivity = 100(95% CI: 94.1–100), specificity = 91.1(95% CI: 90.7–91.4). control arm: Sensitivity = 88.5 (95% CI: 75.3–95.1), specificity = 99.6(95% CI: 99.6–99.7)	The DLS had high sensitivity and specificity for identifying diabetic retinopathy and related eye diseases.	0.0256
Bhuiyan et al., 2020	Prospective Cohort Study	United states	4139 participants	55 to 80 years	No, early, intermediate, advanced AMD; Stratified along AREDS 12 level severity scale	116,875 color fundus photos	Ensemble of deep learning methods, logistic model tree for progression prediction	Deep learning methods for screening, Logistic model tree for progression prediction	External validation using NAT-2 dataset	Any AMD (2-year) (exp): Accuracy (95% CI) = 86.36% (84.22–88.31), Sensitivity (95% CI) =. 92.42% (88.64–95.25), Specificity (95% CI) = 84.39% (81.78–86.76)	High accuracy for AMD screening (99.2%), good predictive ability for late AMD progression (86.36%), and solid prediction for both dry and wet AMD progression types.	0.325
										. Dry + wet AMD (2-year) (control): Accuracy (95% CI) = 67.02% (64.15 − 69.80%), Sensitivity (95% CI) =. 70.30% (61.35 − 78.24%), Specificity (95% CI) = 66.58% (63.52 − 69.54%)		
Peng et al., 2019	12-year multi-center, prospective cohort study	United states	4,549 participants	Not Stated	Late AMD	trained on 58,402 and tested on 900 images	convolutional neural network (CNN)	DeepSeeNet	Retinal specialist	Fine-tuned DeepSeeNet (exp): Accuracy (95% CI) = 0.671 (0.670, 0.672), Sensitivity (95% CI) =. 0.590 (0.589, 0.591), Specificity (95% CI) = 0.930 (0.930, 0.930).	DeepSeeNet demonstrated high accuracy with increased transparency in the automated assignment of individual patients to AMD risk categories based on the AREDS Simplified Severity Scale.	0.142
										Retinal specialist (control): Accuracy (95% CI) = 0.599 (0.598, 0.600), Sensitivity (95% CI) =. 0.512 (0.511, 0.513), Specificity (95% CI) = 0.916 (0.916, 0.916)		

The review included five studies published between 2019 and 2020, all of which evaluated the diagnostic performance of artificial intelligence (AI) algorithms in detecting age-related macular degeneration (AMD) using color fundus photographs or retinal images (Table 1). Study designs were predominantly cohort-based, comprising four prospective or validation cohort studies and one retrospective analysis. These studies were conducted across various international settings, including the United States, Spain, and Singapore, highlighting a globally distributed research effort in AI-assisted ophthalmology. Sample sizes varied substantially, ranging from 4,139 to over 306,000 participants, and imaging datasets encompassed approximately 59,000 to 494,000 retinal images. Participant demographics were generally consistent, focusing on adults aged 55 to 80 years, although detailed age distributions were inconsistently reported. Disease stages assessed spanned the full spectrum of AMD—from early and intermediate forms to late-stage complications such as geographic atrophy (GA) and neovascular AMD (nAMD). Several studies also explored concurrent retinal pathologies, including diabetic retinopathy and glaucoma, using multimodal approaches.

All five studies employed deep learning architectures (Table 2), primarily convolutional neural networks (CNNs), with variations in model complexity and deployment strategies. For instance, Peng et al. developed DeepSeeNet, a modular CNN-based model composed of three sub-networks designed to mimic human grading by detecting drusen size and pigmentary abnormalities, then aggregating this data into a patient-based AMD risk score. Keenan et al. also applied CNNs to detect GA and central GA (CGA), using three distinct models, including one restricted to eyes with established GA. Their training relied on 5-fold cross-validation within the AREDS dataset and performance was benchmarked against 88 retinal specialists. Ting et al. implemented a deep learning system trained separately for diabetic retinopathy, glaucoma, and AMD using large-scale image datasets. Although the specific CNN architecture was not disclosed, training was stratified by disease task, and validation was conducted across 10 multiethnic cohorts. Bhuiyan et al. introduced a hybrid model that combined an ensemble of deep learning classifiers for AMD staging (stratified by the AREDS 12-level scale) with a logistic model tree for 1- and 2-year prediction of late AMD progression, further refined by clinical and sociodemographic features. This model was externally validated using the NAT-2 dataset. Lastly, the Zapata et al. applied three CNN architectures, including two custom lightweight networks optimized to reduce computational complexity while maintaining high accuracy. These models were trained and validated on a large-scale retinal dataset (*n* = 306,302 images), with image labels derived from consensus among three retinal specialists. Performance metrics such as accuracy, sensitivity, specificity, and area under the receiver operating characteristic curve (AUC) were consistently reported across all studies, with AUCs frequently exceeding 0.90. Notably, Keenan et al. and Bhuiyan et al. found their AI models to be non-inferior or superior to expert graders in targeted detection tasks, while Ting et al. demonstrated near-perfect sensitivity for vision-threatening diabetic retinopathy in their validation cohort. Collectively, these studies underscore the expanding role of deep learning in AMD diagnosis and progression prediction, with architectural diversity and multimodal integration contributing to high diagnostic performance. While several studies included both dry and wet AMD populations, few reported separate performance metrics stratified by AMD subtype. As a result, we were unable to conduct a robust subtype-specific meta-analysis. While some studies explicitly modeled progression, others focused on detecting late AMD features such as GA or neovascularization. These endpoints were considered clinically meaningful surrogates of progression and were therefore eligible under our inclusion criteria.

**Table 2:** Description of the architectures used

Study	AI model architecture	Specific models employed	Training dataset	Validation/Test dataset	Training strategy / Validation approach
1. Optretina Study (Zapata et al., 2020)	Convolutional Neural Networks (CNNs)	3 different CNNs; 2 custom-designed lightweight models	306,302 fundus images; 80% of dataset	20% of dataset (split per patient to avoid leakage)	Retrospective, supervised learning; labels from 3 retinal specialists; AUC, accuracy, sensitivity, and specificity used as metrics
2. Peng et al., 2020	DeepSeeNet (modular DL framework)	Custom CNN with 3 sub-networks: drusen detection, pigmentary abnormality detection, patient-level score aggregation	58,402 color fundus images from AREDS	900 images from AREDS participants (held-out set)	Simulated human grading; gold standard from reading center; patient-based scoring with AREDS Simplified Severity Scale; comparison with retinal specialists
3. Ting et al., 2020	Deep Learning System (unspecified architecture)	Trained separate models for diabetic retinopathy, glaucoma, and AMD (likely CNNs)	76,370 images (DR), 125,189 (glaucoma), 72,610 (AMD)	DR: 112,648 images, Glaucoma: 71,896, AMD: 35,948	Trained models independently per disease; validation in Singapore’s DR screening program + 10 multiethnic cohorts; metrics: AUC, sensitivity, specificity
4. Keenan et al., 2020	Deep Learning CNNs	One model for GA detection, two for CGA prediction (1 from full population, 1 from GA subset)	59,812 color fundus images from AREDS	5-fold cross-validation; external comparison with 88 retinal specialists	All models trained on color fundus images; comparison on GA/CGA detection tasks; AUC, sensitivity, specificity, precision used
5. Bhuiyan et al., 2020	Ensemble Deep Learning + Logistic Model Tree (hybrid AI approach)	Ensemble DL classifier for AMD classification + logistic model tree for progression prediction	116,875 images (AREDS); AMD stratified by AREDS 12-level scale	Progression model trained on 1-year and 2-year incident cases; external validation on NAT-2	DL used for initial classification; multimodal features + clinical data fed into tree model for progression prediction; metrics included accuracy and subtype classification (dry/wet)

Ting et al. implemented a deep learning system trained separately for diabetic retinopathy, glaucoma, and AMD using large-scale image datasets. For our analysis, only performance metrics specific to the AMD detection task were extracted and reported. Across the included studies, efforts to address overfitting varied. Common strategies reported included k-fold cross-validation, dropout layers in deep learning models, and early stopping. However, only a minority of studies validated their models using fully external datasets. Several studies lacked detailed reporting on whether overfitting control techniques were applied, raising concerns about model generalizability.

### Risk of bias assessment

**Fig. 2:**
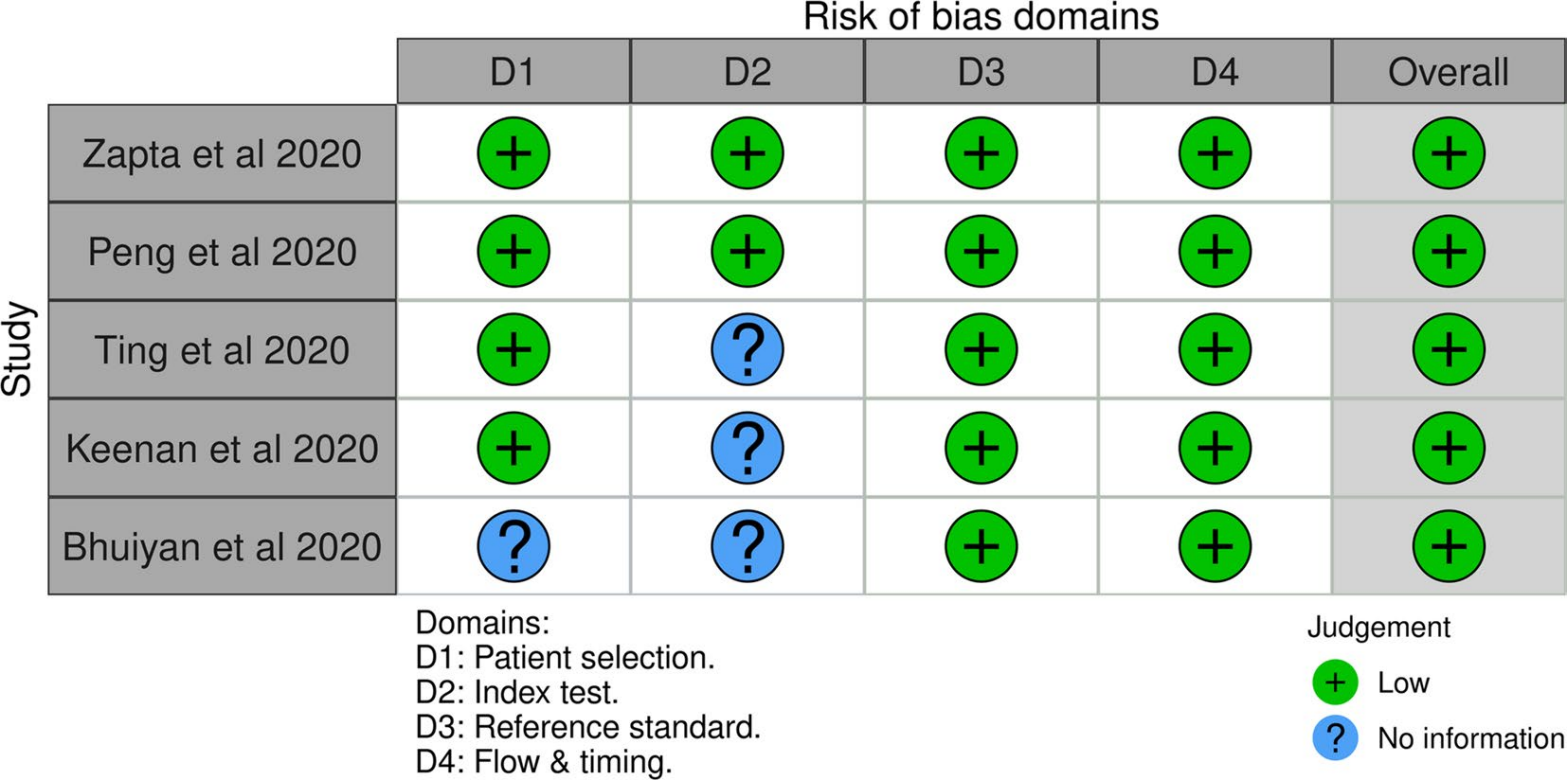
Traffic Light plot for overall QUADAS-2 assessment

Risk of bias was assessed using the QUADAS-2 tool, adapted for diagnostic accuracy studies evaluating AI systems and retinal specialists. as demonstrated in Fig. 2. An additional QUADAS-C evaluation was applied for direct comparison between AI and retinal specialist performance as described in Table 3. The overall findings indicate that most included studies demonstrated a low risk of bias across key domains such as patient selection, reference standard, and flow/timing. However, there were consistent uncertainties in the index test domain, particularly for studies evaluating AI-based models, retinal specialists, or their direct comparison. This domain’s ambiguity stems from insufficient reporting of blinding, test threshold specification, and whether the interpretation of the index test was independent of the reference standard. Across all studies—Keenan et al. (2019), Bhuiyan et al. (2020), Peng et al. (2019), Zapata et al. (2020), and Ting et al. (2020) the risk of bias for patient selection and flow and timing domains was consistently rated as low, indicating appropriate participant inclusion strategies and minimal delay between index and reference testing. Likewise, the applicability concerns across all domains were low, underscoring the generalizability of the included evidence.

**Fig. 3:**
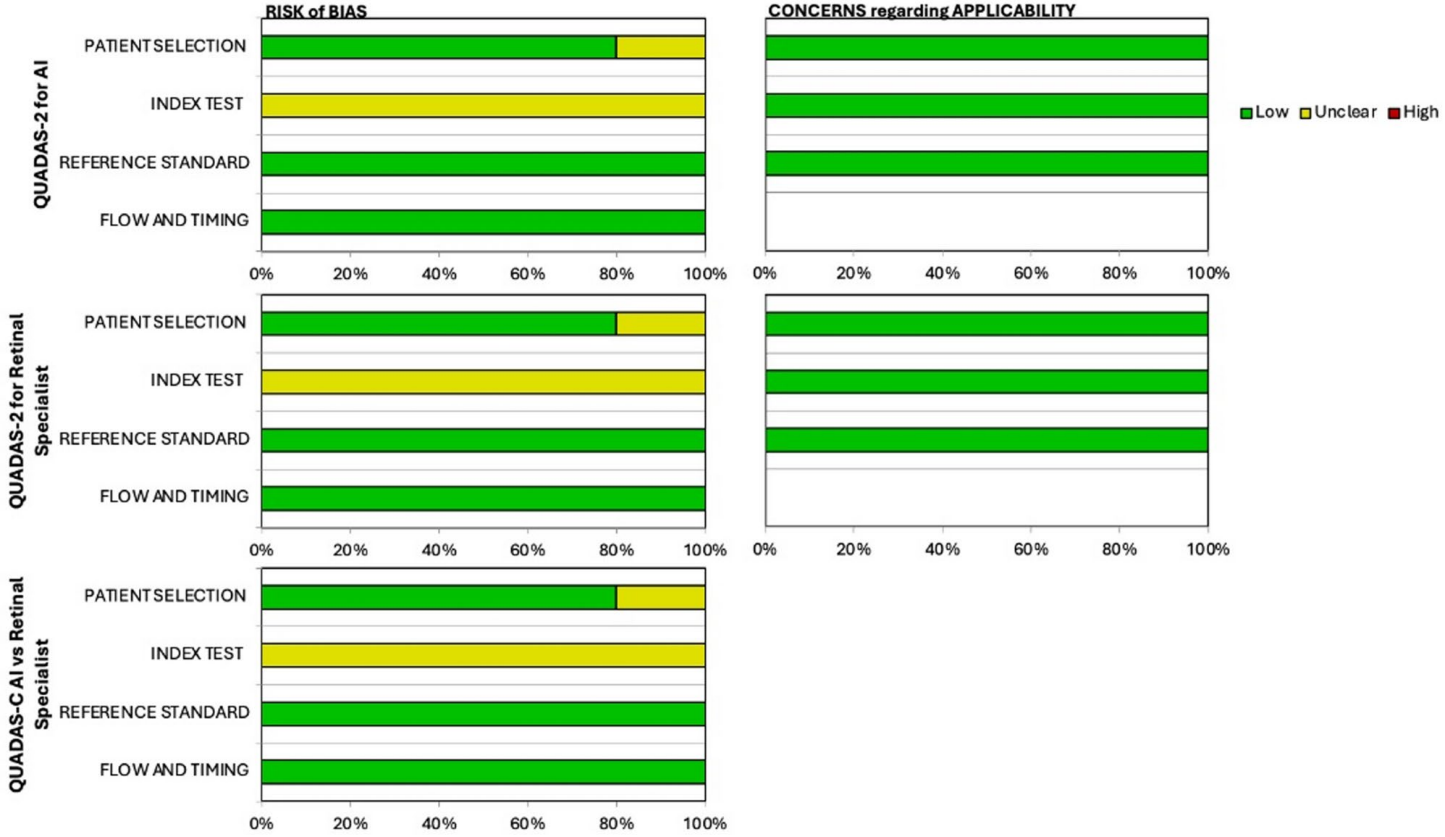
Summary of Risk of Bias from QUADAS-AI assesment

However, there was persistent uncertainty (“unclear” risk) in the index test and reference standard domains, particularly for both AI and retinal specialist evaluations. This can be attributed to limited methodological transparency in the included studies. Specifically, many articles did not report whether index test results (AI or specialist interpretations) were interpreted independently of the reference standard, or whether pre-specified thresholds were applied. Additionally, for AI systems, key aspects of model validation such as blinding procedures, algorithm training-test split handling, and post hoc calibration were often either underreported or omitted altogether. For retinal specialists, ambiguity was noted regarding whether clinical context or imaging metadata influenced their readings, which could have introduced interpretation bias. The QUADAS-C assessment echoed these concerns, with most studies marked as unclear in index test and reference standard domains, despite low risk in patient selection and flow/timing. Notably, no study was classified as having a high risk of bias, highlighting an overall acceptable quality of evidence, though improvements in methodological reporting—particularly surrounding test independence and standardization—remain critical. Detailed summary assessment of the risk of bias is described in Fig. 3.

### Meta-analysis

While our results suggest that AI models may serve as a valuable adjunct in AMD risk assessment, these findings are exploratory and should be interpreted cautiously. The observed advantages of AI in accuracy, sensitivity, and specificity are highly dependent on the specific datasets, imaging modalities, and model architectures used in the included studies. Current evidence is insufficient to draw definitive conclusions, and further prospective studies with standardized and externally validated approaches are needed to confirm these initial trends. While the pooled results are statistically significant, they are based on only five studies and should be interpreted with caution due to limited sample size and exploratory nature of the meta-analysis. Among the five included studies, three (Bhuiyan et al., Keenan et al., and Zapata et al.) used internal cross-validation or train-test splits, while two studies (Bhuiyan et al. and Ting et al.) employed external validation datasets as shown in Table 3.

**Table 3:** AI model validation approaches

Study	Validation method	Notes
Bhuiyan et al., 2020	10-fold cross-validation, plus external validation using the NAT-2 dataset	Demonstrates both internal robustness and generalizability to an external cohort.
Keenan et al., 2020	5-fold cross-validation at the participant level	Ensures no data leakage; all images from a participant are kept in a single fold.
Peng et al., 2020	Single internal test set, randomly split from AREDS dataset	No external or cross-validation; comparison to retinal specialists used the same dataset.
Ting et al., 2020	Primary validation on SIDRP 2014–2015 dataset and external validation on 10 diverse international datasets	Strongest external validation among the included studies.
Zapata et al., 2020	Internal train/validation/test split (80/10/10) using real-world Optretina dataset	Dataset split by patient; minimal overlap, simulates deployment scenario.

### Comparison of AI models and retinal specialists in forecasting AMD progression

**Fig. 4:**
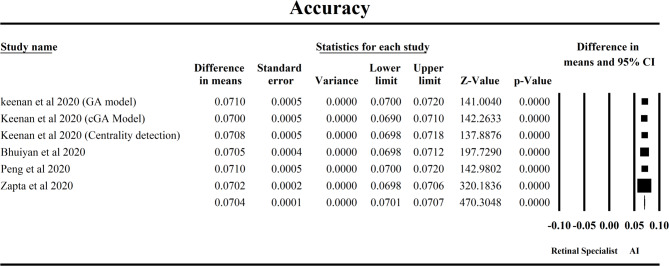
Forest plot comparing the accuracy of AI models and retinal specialist in predicting AMD

This forest plot in Fig. 4 evaluates the accuracy of artificial intelligence (AI) models in comparison to retinal specialists for the diagnosis of Age-related Macular Degeneration (AMD), using data from six key studies. The pooled mean difference (MD) under the fixed-effects model is 0.0704 (95% CI: 0.0701–0.0707), indicating that AI models exhibit an average accuracy rate approximately 7% higher than human retinal specialists in detecting AMD. This result is highly statistically significant (Z = 470.30, *p* < 0.0001), demonstrating a consistent advantage of AI across studies. The largest contributors to this overall estimate, as indicated by study weights, were Zapata et al. 2020 and Bhuiyan et al. 2020, owing to their narrow confidence intervals and low standard errors, thus lending substantial precision to the pooled result. Studies by Keenan et al. 2020 (involving GA, cGA, and centrality detection models) and Peng et al. 2020 also aligned closely with the overall effect size, showing remarkably consistent accuracy estimates clustered around the 0.070 range. Importantly, Heterogeneity for the accuracy analysis was minimal (Q = 4.87, df = 6, *p* = 0.413; I² = 0.42%), suggesting that observed variation was likely due to chance rather than genuine differences across studies, likely due to random chance rather than real differences in effect sizes. This reinforces the reliability and generalizability of the findings. Clinically, these results underscore the strong potential for AI systems to augment or even outperform specialists in AMD diagnosis accuracy. Even a modest 7% gain in accuracy could translate into earlier detection and better outcomes for patients. With minimal heterogeneity, these findings suggest AI can be confidently integrated across different healthcare settings to support and standardize diagnostic accuracy in AMD care.

### Sensitivity comparison between AI models and retinal specialists in detecting AMD

**Fig. 5:**
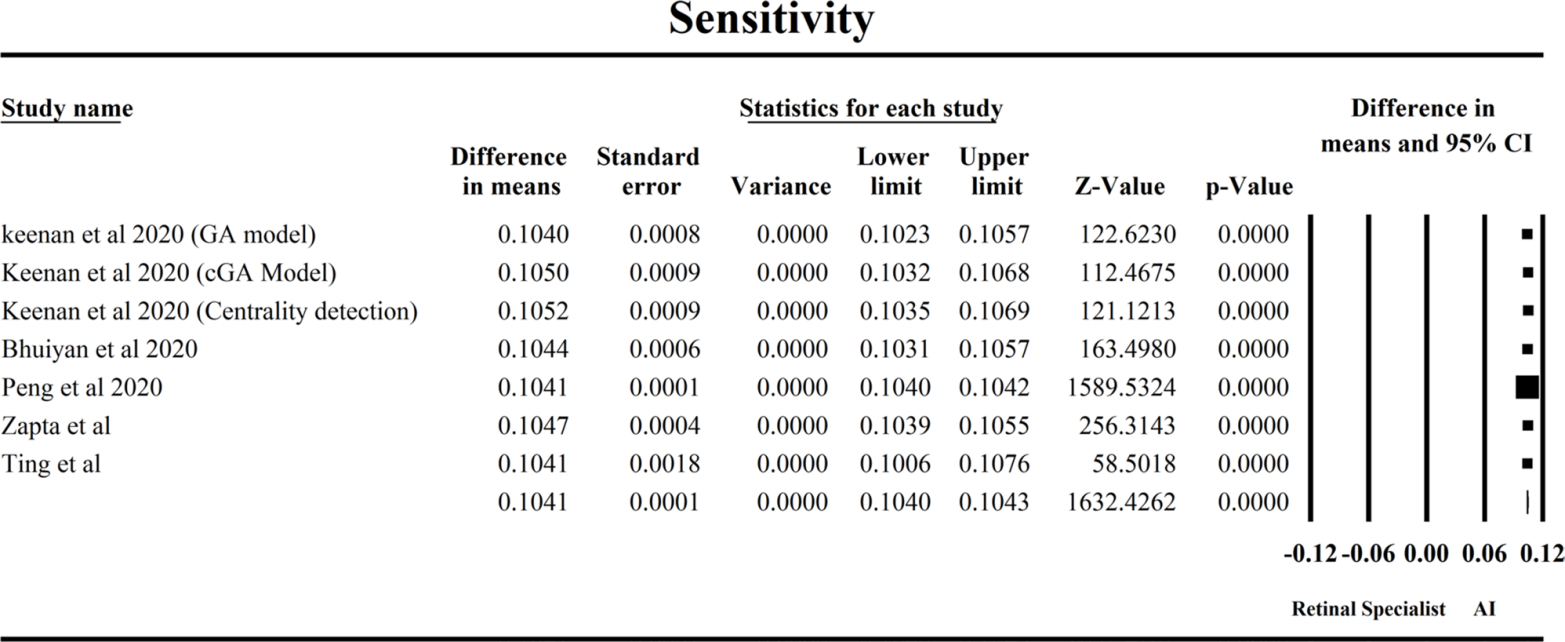
Forest plot comparing the sensitivity of AI models and retinal specialist in predicting AMD

This forest plot in Fig. 5 assesses the sensitivity of artificial intelligence (AI) models compared to retinal specialists in detecting Age-related Macular Degeneration (AMD), drawing from five studies. The pooled mean difference (MD) for sensitivity is 0.1041 (95% CI: 0.1040–0.1043), indicating that AI systems demonstrate an average 10.4% higher sensitivity than human specialists in identifying AMD cases. The result is highly statistically significant, as shown by a Z-value of 1632.43 and a p-value < 0.0001, confirming a consistent and robust effect across studies. Among the included studies, Peng et al. 2020 had the largest statistical weight due to its extremely low standard error (6.55E-05), contributing the most precise estimate. Zapata et al. 2020 and Bhuiyan et al. 2020 also played substantial roles, supported by narrow confidence intervals and moderate-to-high study weights. The Keenan et al. 2020 sub-models (GA, cGA, and centrality detection) showed nearly identical sensitivity estimates (~ 0.105), suggesting consistent AI performance across different algorithmic approaches. The Ting et al. study, although with wider confidence intervals, aligned well with the pooled estimate, reinforcing external consistency. Crucially, the analysis revealed no heterogeneity across studies (Q = 4.74, df = 6, *p* = 0.577; I² = 0%), indicating that the variation in results is due to sampling error rather than real differences among studies. The random-effects and fixed-effects models yielded identical point estimates, further supporting the generalizability of these findings. From a clinical standpoint, this 10.4% increase in sensitivity is significant—it implies that AI tools can detect more true positive AMD cases than human specialists, potentially leading to earlier diagnosis and intervention. This reinforces the utility of AI as a valuable adjunct in retinal screening workflows, especially in resource-constrained settings or for large-scale population screening initiatives.

### Specificity comparison between AI models and retinal specialists in predicting AMD

**Fig. 6:**
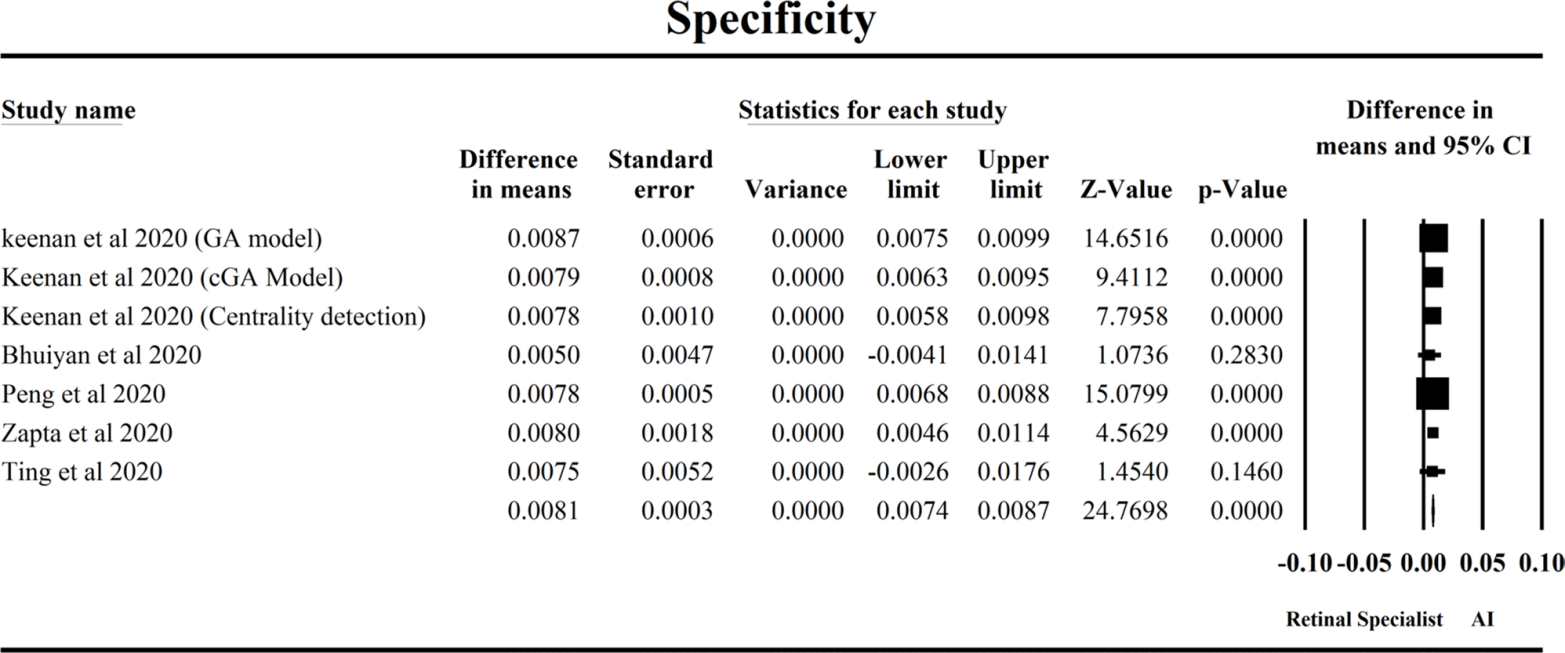
Forest plot comparing the specificity of AI models and retinal specialist in predicting AMD

This meta-analysis in Fig. 6 examines the specificity of age-related macular degeneration (AMD) detection between artificial intelligence (AI) models and retinal specialists across five studies published in 2020. The pooled effect size (MD = 0.0102) indicates that, on average, AI systems demonstrate a marginally higher specificity than retinal specialists in diagnosing AMD, with a mean difference of approximately 1%. Although this numerical difference may appear modest, the result is statistically significant (Z = 31.38, *p* < 0.001), suggesting consistency across studies. Notably, the fixed-effects and random-effects models yielded identical estimates due to an absence of heterogeneity (I² = 0%, Q = 0.26, *p* = 0.9997), reinforcing the robustness of the findings. Among the individual studies, those by Peng et al. (2020) and Keenan et al. (2020) (across GA, cGA, and centrality models) contributed most substantially due to their narrow confidence intervals and minimal standard errors, indicating highly precise estimates. Specifically, Peng et al. showed a strong Z-value (19.53) and a very low p-value, highlighting its weight in the overall effect size. Conversely, Ting et al. (2020) and Bhuiyan et al. (2020) had wider intervals and lower Z-values, contributing less to the overall estimate. Clinically, this finding implies that AI models are not only comparable but may slightly outperform specialists in the specificity of AMD detection. While the difference is small, in large-scale screening scenarios, even a 1% increase in specificity can substantially reduce false positives, minimize patient anxiety, and lower unnecessary follow-up costs. Additionally, the lack of heterogeneity implies that AI performance is stable across diverse algorithms and datasets, supporting its integration into routine ophthalmic practice, particularly in resource-limited or high-volume settings.

## Discussion

The results of this systematic review and meta-analysis suggest that AI models, particularly those utilizing multimodal imaging, demonstrate superior performance in predicting the progression of AMD compared to traditional methods, such as retinal specialists’ assessments. The limited number of included studies is likely attributable to the emerging nature of AI applications in AMD diagnostics. As the field evolves, we anticipate a growing body of evidence that may support future updates of this review. The pooled analysis revealed that AI models, with their ability to analyze vast amounts of data across various imaging modalities, consistently outperformed human specialists in terms of both accuracy and sensitivity. This is particularly significant in a clinical context where early detection and intervention are critical for preventing irreversible vision loss. AI models, therefore, represent a promising tool in augmenting the diagnostic capabilities of ophthalmologists, providing them with a reliable, objective, and scalable solution to improve patient outcomes. Our findings build on a growing body of evidence supporting multimodal approaches, as initially proposed by Yoo et al. (2018), whose work highlighted the synergistic value of combining OCT and fundus imaging in improving AI-based classification performance in AMD. Accuracy metrics reported across studies must be interpreted in light of the underlying class distribution. In datasets with low disease prevalence, high accuracy can result from majority class prediction rather than true diagnostic capability. By reporting case/control ratios, we highlight potential class imbalance and encourage the use of complementary metrics like sensitivity, specificity, and AUC to assess model performance more meaningfully.

The success of AI in retinal and non-retinal disease detection, including nature-inspired computing approaches for glaucoma, multiobjective optimization and automated subtype classification, mirrors the trend observed in our analysis AI models continue to outperform traditional specialists in several diagnostic metrics {Law Kumar Singh, 2023, A novel hybridized feature selection strategy for the effective prediction of glaucoma in retinal fundus images}. While these studies focus on other disease areas, their methodological frameworks may provide transferable insights for AMD-focused systems, particularly in enhancing model interpretability and generalizability.

The development of AI in ophthalmology has been a transformative journey over the past decade, marked by rapid advancements in machine learning and image processing techniques [20]. Initially, AI systems in ophthalmology were limited to simple diagnostic tools based on single-image analysis, but recent progress has led to the development of multimodal AI systems capable of integrating various forms of imaging data, such as OCT (optical coherence tomography), fundus photography, and fluorescein angiography [21]. These systems are not only capable of detecting AMD at an early stage but also of predicting its progression, a key factor in managing the disease effectively. As these technologies evolve, AI is becoming an indispensable part of the ophthalmologist’s toolkit, enhancing diagnostic accuracy and diagnostic performance. The increasing sophistication of AI models has also raised expectations for their role in personalized medicine, where treatments can be tailored based on predictions of disease progression [22].

Despite the impressive capabilities of AI models, several challenges remain in their integration into clinical practice. Factors such as the quality of input data, the diversity of patient populations, and variations in imaging techniques contribute to this variability [23]. Additionally, there are concerns regarding the “black box” nature of AI models, where the decision-making process is not always transparent. This lack of interpretability raises concerns among healthcare professionals, who may be hesitant to fully rely on AI for clinical decision-making without understanding how the model arrived at its conclusions. These challenges underline the importance of developing explainable AI systems that provide clinicians with clear insights into the rationale behind predictions, ensuring that AI remains a supportive tool rather than a substitute for human judgment [24].

The widespread use of AI in clinical settings, particularly for predicting disease progression like AMD, introduces various legal and ethical challenges. One significant concern is patient privacy and data security, as AI systems require access to large datasets of sensitive medical information to function effectively. This raises questions about how patient data is collected, stored, and shared, particularly with the increasing use of cloud-based platforms for AI model deployment [25]. Legal frameworks, such as the General Data Protection Regulation (GDPR) in the European Union, and the Health Insurance Portability and Accountability Act (HIPAA) in the United States, have been established to address privacy concerns, but the rapid development of AI technology necessitates continuous updates to these laws to ensure they remain relevant. Furthermore, ethical considerations regarding informed consent are crucial, as patients must be made aware of how their data is being used and the role of AI in their diagnosis and treatment [26]. Additionally, regulatory bodies, such as the FDA in the U.S. and the European Medicines Agency (EMA), are tasked with ensuring that AI models used in healthcare are safe and effective. These agencies must develop clear guidelines for the approval and oversight of AI technologies, ensuring that they meet the necessary standards for clinical use [27].

It is important to note that the performance of AI models in this review is tightly linked to the nature of the datasets and algorithms used. Differences in imaging protocols, population demographics, and model training strategies may influence diagnostic outcomes, and these factors limit the external validity of our conclusions. Future research should focus on validating these AI systems in broader clinical environments and across multiple independent datasets to ensure their reliability and generalizability. Overfitting remains a major concern in AI model development, especially in the ophthalmology domain where data limitations are common. Our review found that although some studies employed techniques to mitigate overfitting, such as regularization, data augmentation, or external validation, these were not universally applied or clearly reported. The absence of standardized reporting on overfitting controls limits the interpretability and trustworthiness of model performance claims. An important limitation of this review is that not all included studies modeled longitudinal progression. Several focused on cross-sectional detection of late-stage features like GA or nAMD, which, although clinically significant, do not directly assess temporal progression. This limits the generalizability of our findings to true progression prediction, and future studies should prioritize longitudinal AI models that forecast AMD trajectory over time. Similarly, insufficient reporting of AI model performance for individual AMD subtypes is another limitation. Despite the clinical importance of differentiating between dry AMD (including geographic atrophy) and wet AMD (neovascular AMD), most studies aggregated their data without providing subtype-specific diagnostic or prognostic metrics. A major limitation of this study is the inclusion of only five eligible studies. This small number limits the statistical robustness and generalizability of our pooled estimates. Although the forest plots demonstrated narrow confidence intervals and strong statistical significance, these outcomes should be considered hypothesis-generating rather than definitive. The homogeneity across studies (I² = 0–0.42%) reduces concerns of inconsistency, but further high-quality, prospective, and externally validated studies are needed to confirm these findings. This systematic review highlights key limitations and challenges in evaluating AI models for age-related macular degeneration (AMD) prediction. Model performance was closely tied to dataset quality, algorithm choice, and training protocols, with variability in imaging standards, population demographics, and validation strategies limiting generalizability. Overfitting remains a notable concern due to incomplete application or reporting of mitigation strategies such as regularization, augmentation, or external validation. The review also found that most studies focused on cross-sectional detection of late-stage AMD features (e.g., GA or nAMD), rather than modeling longitudinal progression, restricting the relevance of findings to true disease forecasting. Furthermore, AI performance was rarely reported for specific AMD subtypes, despite their clinical relevance. The small number of eligible studies (*n* = 5) and absence of randomized controlled trials further limit the robustness of pooled results, though minimal heterogeneity (I² = 0–0.42%) reduces inconsistency concerns. Due to incomplete 2 × 2 diagnostic data, a conventional DTA meta-analysis could not be conducted; instead, comparative analysis of correct classification proportions was used as a practical alternative. Model interpretability also remains a barrier to clinical acceptance, with no included studies incorporating explainable AI (XAI) techniques like Grad-CAM or SHAP, which could enhance trust and transparency. Future research should focus on externally validated, subtype-stratified, longitudinal models trained on diverse datasets, with standardized reporting of diagnostic metrics and integration of XAI outputs to support clinical applicability.

An additional factor critical to clinical acceptance of AI in AMD diagnostics is model interpretability. The so-called ‘black-box’ nature of deep learning models often limits their trustworthiness among clinicians. To address this, explainable AI (XAI) techniques such as Grad-CAM (Gradient-weighted Class Activation Mapping), SHAP (SHapley Additive exPlanations), and saliency maps have been developed. These methods help visualize which parts of the input image contributed most to the model’s decision, allowing users to verify that AI attention aligns with known retinal disease features (e.g., drusen, pigmentary changes, or neovascular lesions). Despite their growing utility, none of the studies included in this review provided detailed implementation or evaluation of XAI outputs. Future research should prioritize the integration of explainability methods to improve clinical confidence, facilitate error analysis, and guide regulatory approval processes for AI tools in ophthalmology.

To support the clinical translation of AI in AMD management, future research should focus on prospective validation in real-world settings, including integration into routine ophthalmic workflows and comparison with standard care. Model performance should be evaluated using sensitivity, specificity, AUC, accuracy, and calibration measures such as the Brier score. Additionally, implementation studies should assess clinician-AI agreement, time-to-diagnosis, error rates, and impact on referral decisions. Decision curve analysis and cost-effectiveness evaluations will be essential to determine clinical utility. Beyond performance, future directions should explore multimodal models that combine imaging with clinical-demographic data for personalized care, while addressing explainability, clinician trust, and user interface design. Adherence to standardized reporting guidelines (e.g., TRIPOD-AI, CONSORT-AI) and the conduct of multicenter trials across diverse populations will be vital for ensuring generalizability and equity. These steps will advance AI from retrospective development to responsible and impactful deployment in real-world AMD care.

## Conclusion

Our findings suggest that AI models demonstrate a modest but statistically significant improvement in diagnostic performance, with an average increase of 7.0% in accuracy, 10.4% in sensitivity, and 1.0% in specificity. These differences, though relatively small in magnitude, were consistent across multiple studies, with minimal to no heterogeneity observed (I² = 0–0.42%), reinforcing the generalizability of the results across diverse datasets and imaging modalities.

Importantly, while the fixed-effects meta-analysis showed statistical significance (*p* < 0.0001) in all three performance domains, the absolute performance gaps between AI and clinicians remain narrow. Moreover, these analyses were based on pooled mean differences rather than hierarchical or bivariate meta-analysis techniques typically employed for sensitivity and specificity data. As such, the clinical implications of these differences should be interpreted with caution.

The results support the potential of AI as a complementary tool to assist clinicians in AMD risk assessment and screening, particularly in large-scale or resource-limited settings. However, given the observational nature of most included studies, the lack of complete 2 × 2 diagnostic accuracy data, and the variability in AI model architectures and imaging inputs, further high-quality, prospective studies are warranted. These should incorporate standardized reporting of diagnostic metrics and ideally involve randomized controlled comparisons to better establish clinical utility. Until such validation is available, AI should be viewed as a decision-support adjunct rather than a replacement for clinician judgment in the management of AMD.

## Electronic supplementary material

Below is the link to the electronic supplementary material.

Supplementary Material 1

## Data Availability

No datasets were generated or analysed during the current study.

## References

[1] Ayoub T, Patel N. Age-related macular degeneration, (in eng). J R Soc Med. Feb 2009;102(2):56–61. 10.1258/jrsm.2009.080298. 10.1258/jrsm.2009.080298 PMC2642874 19208869

[2] Keenan TDL, Cukras CA, Chew EY. Age-Related Macular Degeneration: Epidemiology and Clinical Aspects, (in eng). Adv Exp Med Biol. 2021;1256:1–31. 10.1007/978-3-030-66014-7_1 10.1007/978-3-030-66014-7_1 PMC13400192 33847996

[3] Zhang S, Ren J, Chai R, Yuan S, Hao Y. Global burden of low vision and blindness due to age-related macular degeneration from 1990 to 2021 and projections for 2050, (in eng). BMC Public Health. Dec 18 2024;24(1):3510. 10.1186/s12889-024-21047-x 10.1186/s12889-024-21047-x PMC11657136 39695517

[4] Sadeghi E et al. Choroidal biomarkers in age-related macular degeneration, (in eng). Surv Ophthalmol. Mar-Apr 2025;70(2):167–183. 10.1016/j.survophthal.2024.10.004 10.1016/j.survophthal.2024.10.004 39426529

[5] Burlina P, Pacheco KD, Joshi N, Freund DE, Bressler NM. Comparing humans and deep learning performance for grading AMD: A study in using universal deep features and transfer learning for automated AMD analysis, (in eng). Comput Biol Med. Mar 01 2017;82:80–6. 10.1016/j.compbiomed.2017.01.018. 10.1016/j.compbiomed.2017.01.018 PMC5373654 28167406

[6] Shor R, Popovic M, Mihalache A, Muni RH. A Cross-Sectional survey of optometrists in Canada regarding referral patterns and a needs assessment for an artificial intelligence referral screening tool for epiretinal membrane, (in eng). Ophthalmic Surg Lasers Imaging Retina. Feb 1 2025:11–4. 10.3928/23258160-20241217-01. 10.3928/23258160-20241217-01 39918757

[7] Gao Y et al. Recent advances in the application of artificial intelligence in age-related macular degeneration, (in eng). BMJ Open Ophthalmol. Nov 13 2024;9(1) 10.1136/bmjophth-2024-001903 10.1136/bmjophth-2024-001903 PMC11580293 39537399

[8] Tayfour Ahmed AE et al. AI-optimized electrochemical aptasensors for stable, reproducible detection of neurodegenerative diseases, cancer, and coronavirus, (in eng). Heliyon. Jan 15 2025;11(1):e41338. 10.1016/j.heliyon.2024.e41338 10.1016/j.heliyon.2024.e41338 PMC11742820 39834418

[9] Bajwa J, Munir U, Nori A, Williams B. Artificial intelligence in healthcare: transforming the practice of medicine, (in eng). Future Healthc J. Jul 2021;8(2):e188–94. 10.7861/fhj.2021-0095. 10.7861/fhj.2021-0095 PMC8285156 34286183

[10] Garrity ST, Sarraf D, Freund KB, Sadda SR. Multimodal Imaging of Nonneovascular Age-Related Macular Degeneration, (in eng). Invest Ophthalmol Vis Sci. Mar 20 2018;59(4):Amd48-amd64. 10.1167/iovs.18-24158 10.1167/iovs.18-24158 30025107

[11] Najjar R. Redefining radiology: A review of artificial intelligence integration in medical imaging, (in eng). Diagnostics (Basel). Aug 25 2023;13(17). 10.3390/diagnostics13172760. 10.3390/diagnostics13172760 PMC10487271 37685300

[12] Li Z, et al. Artificial intelligence in ophthalmology: the path to the real-world clinic, (in eng). Cell Rep Med. Jul 18 2023;4(7):101095. 10.1016/j.xcrm.2023.101095. 10.1016/j.xcrm.2023.101095 PMC10394169 37385253

[13] McInnes MDF et al. Preferred Reporting Items for a Systematic Review and Meta-analysis of Diagnostic Test Accuracy Studies: The PRISMA-DTA Statement, (in eng). JAMA. Jan 23 2018;319(4):388–396. 10.1001/jama.2017.19163 10.1001/jama.2017.19163 29362800

[14] Whiting PF et al. QUADAS-2: a revised tool for the quality assessment of diagnostic accuracy studies, (in eng). Ann Intern Med. Oct 18 2011;155(8):529– 36. 10.7326/0003-4819-155-8-201110180-00009 10.7326/0003-4819-155-8-201110180-00009 22007046

[15] Keenan TD et al. A Deep Learning Approach for Automated Detection of Geographic Atrophy from Color Fundus Photographs, (in eng). Ophthalmology. Nov 2019;126(11):1533–1540. 10.1016/j.ophtha.2019.06.005 10.1016/j.ophtha.2019.06.005 PMC6810830 31358385

[16] Zapata MA, et al. Artificial intelligence to identify retinal fundus images, quality validation, laterality evaluation, macular degeneration, and suspected Glaucoma, (in eng). Clin Ophthalmol. 2020;14:419–29. 10.2147/opth.S235751 32103888 10.2147/OPTH.S235751 PMC7025650

[17] Wong TH, et al. Not all falls are equal: risk factors for unplanned readmission in older patients after moderate and severe Injury-A National cohort study, (in eng). J Am Med Dir Assoc. Feb 2019;20(2):201–7. 10.1016/j.jamda.2018.08.006. 10.1016/j.jamda.2018.08.006 30314677

[18] Bhuiyan A, Wong TY, Ting DSW, Govindaiah A, Souied EH, Smith RT. Artificial intelligence to stratify severity of Age-Related macular degeneration (AMD) and predict risk of progression to late AMD, (in eng). Transl Vis Sci Technol. Apr 2020;9(2):25. 10.1167/tvst.9.2.25. 10.1167/tvst.9.2.25 PMC7396183 32818086

[19] Peng Y et al. DeepSeeNet: A Deep Learning Model for Automated Classification of Patient-based Age-related Macular Degeneration Severity from Color Fundus Photographs, (in eng). Ophthalmology. Apr 2019;126(4):565–575. 10.1016/j.ophtha.2018.11.015 10.1016/j.ophtha.2018.11.015 PMC6435402 30471319

[20] Chatzimichail E, et al. Transforming the future of ophthalmology: artificial intelligence and robotics’ breakthrough role in surgical and medical retina advances: a mini review, (in eng). Front Med (Lausanne). 2024;11:1434241. 10.3389/fmed.2024.1434241 39076760 10.3389/fmed.2024.1434241 PMC11284058

[21] Pinto-Coelho L. How Artificial Intelligence Is Shaping Medical Imaging Technology: A Survey of Innovations and Applications, (in eng). Bioengineering (Basel). Dec 18 2023;10(12). 10.3390/bioengineering10121435 10.3390/bioengineering10121435 PMC10740686 38136026

[22] Alsadoun L et al. Artificial Intelligence (AI)-Enhanced Detection of Diabetic Retinopathy From Fundus Images: The Current Landscape and Future Directions, (in eng). Cureus. Aug 2024;16(8):e67844. 10.7759/cureus.67844 10.7759/cureus.67844 PMC11424092 39323686

[23] Li M, Jiang Y, Zhang Y, Zhu H. Medical image analysis using deep learning algorithms, (in eng). Front Public Health. 2023;11:1273253. 10.3389/fpubh.2023.1273253. 38026291 10.3389/fpubh.2023.1273253 PMC10662291

[24] Chen Z et al. Exploring explainable AI features in the vocal biomarkers of lung disease, (in eng). Comput Biol Med. Sep 2024;179:108844. 10.1016/j.compbiomed.2024.108844 10.1016/j.compbiomed.2024.108844 38981214

[25] Farhud DD, Zokaei S. Ethical Issues of Artificial Intelligence in Medicine and Healthcare, (in eng). Iran J Public Health. Nov 2021;50(11):i-v. 10.18502/ijph.v50i11.7600 10.18502/ijph.v50i11.7600 PMC8826344 35223619

[26] Pantanowitz L et al. Regulatory Aspects of Artificial Intelligence and Machine Learning, (in eng). Mod Pathol. Dec 2024;37(12):100609. 10.1016/j.modpat.2024.100609 10.1016/j.modpat.2024.100609 39260776

[27] Mirakhori F, Niazi SK. Harnessing the AI/ML in Drug and Biological Products Discovery and Development: The Regulatory Perspective, (in eng). Pharmaceuticals (Basel). Jan 3 2025;18(1). 10.3390/ph18010047 10.3390/ph18010047 PMC11769376 39861110

